# Supplementary material for: A network dynamical simulation model for the study of antibiotic resistance in microbial communities
Source: Gut Microbes. 2026 Sep 19;18(1):2734703. doi: 10.1080/19490976.2026.2734703 (PMC13596919; doi:10.1080/19490976.2026.2734703)
Supplement: Supplementary file 1 [file KGMI_A_2734703_SM9053.zip › supplementary_file_1/Figures/empirical_resistance_timeseries_k0_5_seed_high_degree_n_347_tx_0_T_21.pdf]

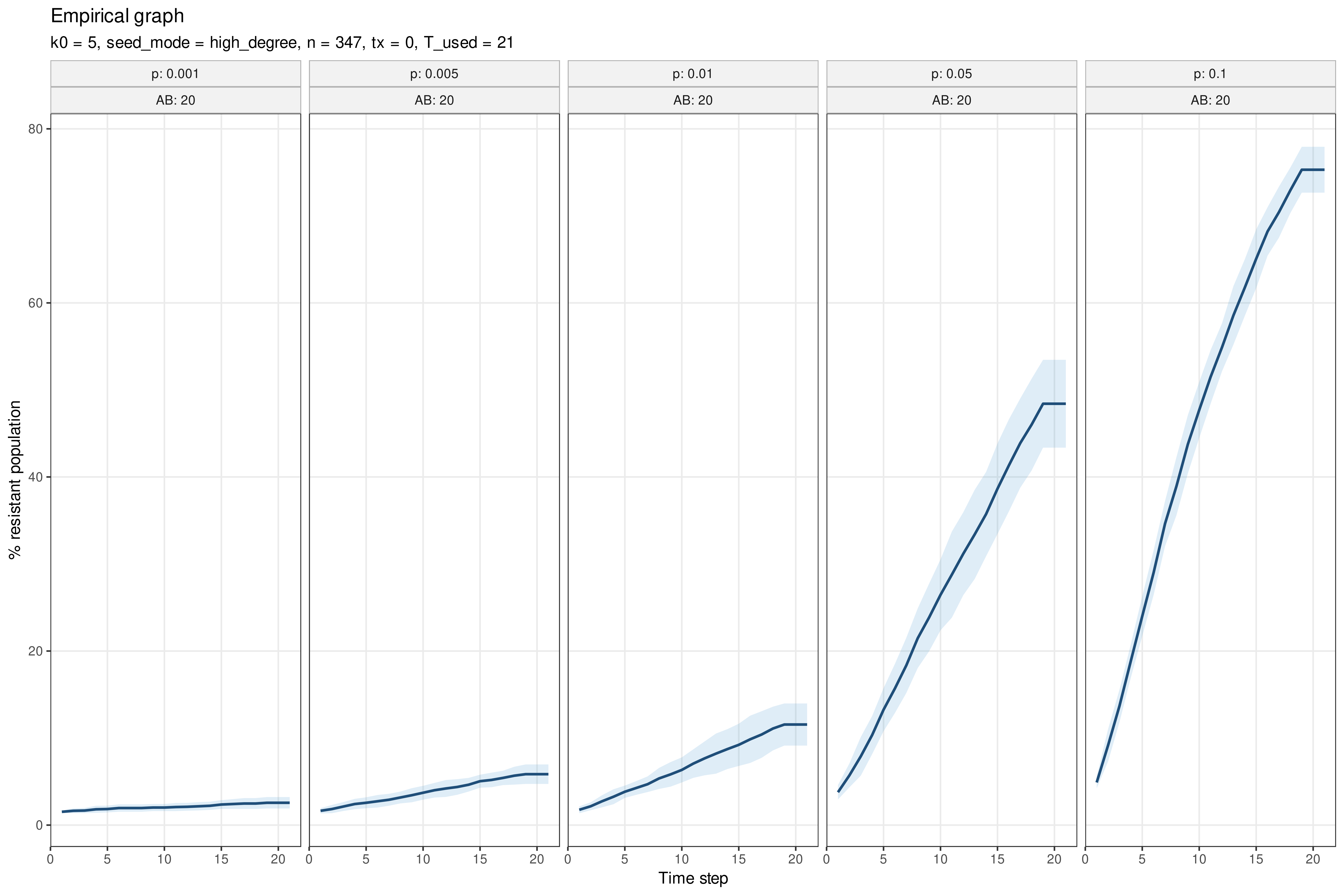

Empirical graph
k0 = 5, seed_mode = high_degree, n = 347, tx = 0, T_used = 21
p: 0.001
p: 0.005
p: 0.01
p: 0.05
p: 0.1
AB: 20
AB: 20
AB: 20
AB: 20
AB: 20
% resistant population
0
20
40
60
80
0
5
10
15
20
Time step
